# T‐cell egress from the thymus: Should I stay or should I go?

**DOI:** 10.1002/JLB.1MR1217-496R

**Published:** 2018-02-27

**Authors:** Kieran D. James, William E. Jenkinson, Graham Anderson

**Affiliations:** ^1^ Institute of Immunology and Immunotherapy College of Medical and Dental Sciences Medical School University of Birmingham Birmingham UK

**Keywords:** chemokine, migration, thymocyte

## Abstract

T‐cells bearing the αβTCR play a vital role in defending the host against foreign pathogens and malignant transformation of self. Importantly, T‐cells are required to remain tolerant to the host's own cells and tissues in order to prevent self‐reactive responses that can lead to autoimmune disease. T‐cells achieve the capacity for self/nonself discrimination by undergoing a highly selective and rigorous developmental program during their maturation in the thymus. This organ is unique in its ability to support a program of T‐cell development that ensures the establishment of a functionally diverse αβTCR repertoire within the peripheral T‐cell pool. The thymus achieves this by virtue of specialized stromal microenvironments that contain heterogeneous cell types, whose organization and function underpins their ability to educate, support, and screen different thymocyte subsets through various stages of development. These stages range from the entry of early T‐cell progenitors into the thymus, through to the positive and negative selection of the αβTCR repertoire. The importance of the thymus medulla as a site for T‐cell tolerance and the exit of newly generated T‐cells into the periphery is well established. In this review, we summarize current knowledge on the developmental pathways that take place during αβT‐cell development in the thymus. In addition, we focus on the mechanisms that regulate thymic egress and contribute to the seeding of peripheral tissues with newly selected self‐tolerant αβT‐cells.

## Abstract 2

Review on thymic microenvironments regulation of thymocyte maturation and egress of mature self‐tolerant T cells.

**Abbreviations**
3PP: 3rd pharyngeal pouch
Aire: autoimmune regulator
APECED: autoimmune‐polyendocrinopathy‐candidiasis‐ectodermal dystrophy
Cers2: ceramide synthase 2
CMJ: corticomedullary junction
cTEC: cortical thymic epithelial cell
CTS: Cataract Shionogi
ETP: early T‐cell progenitor
Fezf2: Fez family zinc‐finger 2
Foxo1: Forkhead box protein O1
Klf2: Krüpple‐like factor 2
LPP3: lipid phosphate phosphatase 3
LTβR: lymphotoxin beta receptor
M1: mature 1
M2: mature 2
mTEC: medullary thymic epithelial cell
Ptcd: peripheral T‐cell deficiency
PVS: perivascular space
RTE: recent thymic emigrant
S1P: sphingosine‐1‐phosphate
S1PR1: sphingosine‐1‐phosphate receptor 1
SGPL: S1P lyase
SM: semimature
SPHK: sphingosine kinase
Spns2: spinster homolog 2
TEC: thymic epithelial cell
TRA: tissue‐restricted antigen
T‐reg: regulatory T‐cell
TSP: thymus settling progenitor

## 1 INTRODUCTION

The murine thymus appears during early stages of embryogenesis, arising from the 3rd pharyngeal pouch (3PP) and initially consisting of an endodermal‐derived epithelial rudiment surrounded by neural crest‐derived mesenchyme.^1^, ^2^ Development of the thymus is dependent on the transcription factor Foxn1, which plays a key role in multiple aspects of thymic epithelial cell (TEC) regulation, including their differentiation, proliferation, and formation of the 3‐dimensional TEC network characteristic of thymic parenchyma.^3^, ^4^, ^5^ The development of many cell subtypes of the thymic microenvironment is dependent on bidirectional signaling between stromal cells and developing thymocytes. An example of this is the signaling between TECs and thymocytes often referred to as “cross‐talk” whereby the development of each population is interdependent on interactions with each other.

**Figure 1:**
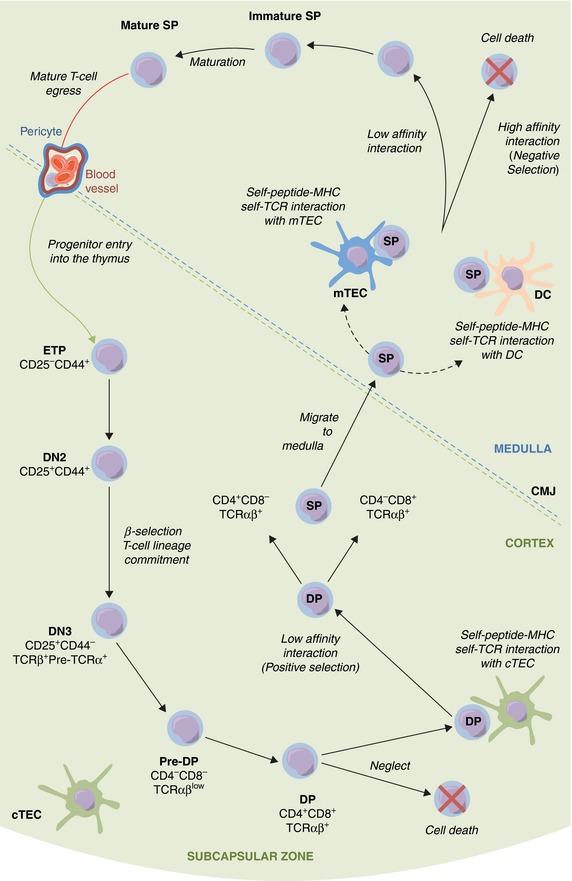
**Pathways in intrathymic T‐cell development**. T‐cell development in the thymus involves a complex series of stages that involve the stepwise migration of developing thymocytes through cortical and medullary thymic microenvironments. At the corticomedullary junction (CMJ), T‐cell progenitors enter the thymus via blood vessels surrounded by pericytes, and develop into CD25^−^CD44^+^CD117^+^ early T‐cell progenitors (ETPs). In the cortex, ETPs progress through CD25/CD44 DN stages, which involves migration along a cellular matrix comprised of VCAM‐1‐expressing cTEC. Cortex‐resident DP thymocytes then express the αβTCR, and undergo positive selection, when successful low affinity αβTCR interactions between DP thymocytes and cTEC occur. This generates CD4^+^ and CD8^+^ SP thymocytes, which migrate to the medulla where negative selection takes place of those cells expressing TCRs that bind self‐peptide‐self‐MHC complexes with high affinity. Following intrathymic selection, SP thymocytes undergo final intrathymic maturation, acquire egress‐competence and exit the thymus via blood vessels at the CMJ

Intrathymic T‐cell development occurs in a step‐wise manner, where immature thymocytes undergo progressive maturation within thymic microenvironments (Fig. 1). Unlike the bone marrow, the thymus does not contain a local pool of hematopoietic stem cells. Consequently, T‐cell development is dependent on the colonization of the thymus by blood‐borne progenitor cells that initially arise from remote microenvironments.^6^ Hematopoietic progenitor entry occurs in waves during both prenatal development and adulthood. During fetal early stages, progenitors are mainly sourced from the liver and enter the early thymic rudiment by migrating through the surrounding mesenchyme layer and mainly give rise to multiple waves of invariant γδT‐cells as well as αβT‐cells.^3^, ^7^, ^8^, ^9^, ^10^ In the postnatal and adult, thymus αβT‐cell development dominates and progenitors are sourced from the bone marrow and enter the thymus via blood vessels at the corticomedullary junction (CMJ).^9^, ^10^, ^11^ Thus, although the thymus produces T‐cells throughout life, there are qualitative differences in both the lymphoid progenitors that are recruited to the thymus and the types of T‐cell they generate. During steady‐state T‐cell development in the adult mouse, the progenitor cell(s) that represent thymus settling progenitors (TSPs) and undergo thymus colonization remain poorly understood.^12^ This is likely at least in part due to the very small number of these cells that exist within the adult thymus, as well as the T‐cell developmental capacity of multiple bone marrow progenitors that can colonize the thymus in a variety of experimental settings. However, downstream of TSP, intrathymic early T‐cell progenitors (ETPs) and their progeny have been well defined. ETPs, identified by a CD4^−^CD8^−^CD25^−^CD44^+^CD117^+^ phenotype, have multilineage potential, as T‐cell lineage commitment does not occur until progeny downstream of the ETPs.^13^ However in contrast to the idea that thymus colonizing cells have multilineage potential, the use of IL‐7Rα^cre^ fate mapping by Schlenner et al.^14^ showed that the vast majority of thymocytes had developed from an *Il7r* expressing pathway, suggesting a lymphoid bias in the progenitors that enter the thymus. ETPs develop into CD4^−^CD8^−^CD25^+^CD44^+^ DN2 thymocytes and, following a period of proliferation, these cells down‐regulate CD44 and CD117, developing into CD4^−^CD8^−^CD25^+^CD44^−^ DN3 cells which have lost B‐cell potential but still retain NK‐cell, dendritic cell (DC), and T‐cell lineage potential.^15^, ^16^, ^17^ DN3 thymocytes undergo TCRβ rearrangement, and in‐frame rearrangement of TCRβ chains subsequently results in the expression of a pre‐TCR complex enabling DN3 thymocytes to undergo β‐selection and progress to the CD4^+^CD8^+^ DP stage, where TCRα rearrangements occur and allow expression of the αβTCR complex. CD4^+^CD8^+^ DP thymocytes reside in the cortex, have a 3–4 day lifespan, and die by neglect in the absence of αβTCR signals.^18^ As TCR gene rearrangements occur randomly, the αβTCR repertoire is highly diverse and must be appropriately screened for its ability to recognize self‐peptide/self‐MHC complexes. The first step in this process is termed positive selection, a process in which DP thymocytes expressing an αβTCR that recognizes and binds to self‐peptide/self‐MHC complexes presented by cortical TECs (cTECs) above a minimum recognition threshold triggers their further differentiation.^19^, ^20^ Indeed, DP thymocytes are programmed for cell death by default and it is the interaction between TCR and self‐peptide self‐MHC complexes that induces TCR signaling that promotes survival and differentiation.^21^ Positive selection of DP thymocytes also results in commitment and differentiation into either CD4^+^CD8^−^ SP4 or CD4^−^CD8^+^ SP8 thymocytes, recognizing MHC Class II or Class I, respectively.^22^ Exit from the cortex is determined by the upregulation of CCR7^23^, ^24^ by positively selected thymocytes and expression of the semaphorin 3E receptor PlexinD1.^25^ This enables newly selected cells to migrate away from CCL25 expressing cortical microenvironments toward the thymus medulla, a region rich in the CCR7‐ligands CCL19 and CCL21 that are expressed by multiple stromal cells including medullary thymic epithelium (mTEC). As such, the thymus medulla acts as a repository for newly produced CD4^+^ and CD8^+^ thymocytes capable of self‐MHC recognition. Importantly, interactions between these semimature (SM) thymocytes and their surrounding stromal microenvironments ensure effective T‐cell tolerance is achieved via the removal of self‐reactive thymocytes and Foxp3^+^ regulatory T‐cell development, as well as the regulated exit of mature self‐tolerant T‐cells from the thymus.

## 2 THYMUS MEDULLA ORGANIZATION FOR T‐CELL TOLERANCE AND POSTSELECTION MATURATION

Thymic microenvironments contain epithelial cells, and are organized into distinct cortex and the medulla areas. The developmental transitions that thymocytes undergo are regulated by signals from the microenvironments that they inhabit, with different signals and cell types being present in distinct regions of the thymus. For example, cTECs within the cortex of the thymus regulate the proliferation and differentiation of DN and DP thymocytes through their production of cytokines (e.g., IL‐7), chemokines (e.g., CXCL12), and expression of Notch ligands (e.g., DLL4).^26^, ^27^, ^28^, ^29^

Similarly, within the thymic medulla, mTECs are specialized for specific stages of thymocyte development. For example, mTECs are essential regulators of tolerance induction via both negative selection and Foxp3^+^ natural regulatory T‐cell (nT‐Reg) development. The importance of mTEC for T‐cell tolerance is highlighted in mice that lack organized medullary structures, including mTEC‐deficient Relb^−/−^ mice, and mice lacking members of the TNFR superfamily (e.g., CD40, RANK, LTβR), all of which show signs of T‐cell‐mediated autoimmunity.^30^, ^31^, ^32^, ^33^, ^34^ Negative selection is thought to play a key role in establishing central tolerance, and involves the clonal deletion of autoreactive T‐cells to limit their escape into peripheral tissues. The process of negative selection occurs through apoptosis of thymocytes that bear high affinity TCRs for self and therefore bind strongly to self‐peptide MHC complexes triggering strong TCR signals. In addition, lineage diversion of CD4^+^ SP thymocytes, that involves expression of the master transcription factor Foxp3, results in the formation of nT‐Reg that leave the thymus and populate peripheral tissues to limit functional responses of autoreactive T‐cells that have escaped negative selection.^35^ mTECs are highly specialized in their ability to enforce both thymic tolerance mechanisms. This is at least in part through their ability to ectopically express tissue‐restricted antigens (TRAs). TRAs are self‐proteins that are usually restricted to functionally distinct cells within peripheral tissues, however mTECs are able to ectopically generate such proteins and present them as peptides to developing thymocytes. Two key regulators have been identified within mTECs to regulate the expression of TRAs, the autoimmune regulator (Aire) and Fezf2. Aire is expressed within a specific subpopulation of mTECs and has been shown to be functionally important in both humans and mice, with human patients that carry a genetic mutation of Aire suffering from autoimmune‐polyendocrinopathy‐candidiasis‐ectodermal dystrophy (APECED) and Aire‐deficient mice exhibiting a similar autoimmune disorder.^36^, ^37^, ^38^ This is due to the requirement for Aire for the deletion of specific self‐reactive clones of T‐cells carrying TCRs specific to Aire‐dependent genes and the development of nT‐Reg.^38^, ^39^ However not all TRAs are Aire dependent, as mTECs are able to express some TRAs independently of Aire. mTECs express Fez family zinc‐finger 2 (Fezf2), which has been shown to promote promiscuous gene expression of Aire‐independent TRAs.^40^ Both Aire‐deficient mice and mice lacking Fezf2 in TECs have been shown to exhibit autoimmune deficiencies, highlighting these regulators of TRA expression as key regulators of central tolerance induction.^36^, ^37^, ^38^, ^40^ Although the expression of Fezf2 in mTEC was thought to be regulated by lymphotoxin beta receptor (LTβR)‐signaling, a known regulator of mTEC development, further analysis of LTβR‐deficient mice revealed continued expression of both Fezf2 as well as Aire in mTEC.^30^, ^32^, ^40^, ^41^ Interestingly, the RANK‐RANKL signaling axis initially shown to control the development of Aire^+^ mTEC was recently found to additionally regulate development of Fezf2^+^ mTEC, highlighting a common developmental signaling pathway in the formation of medullary microenvironments essential for central tolerance.^32^, ^34^, ^42^ Thus, the mTEC population as a whole expresses a vast array of self‐antigens, and their presentation either directly or indirectly via cross‐transfer to DC within the medulla effectively screens the newly selected TCR repertoire for self‐reactivity.

The ability of the thymic medulla to effectively support T‐cell tolerance relies on the regulated colocalization of positively selected SP thymocytes bearing the chemokine receptor CCR7, and mTECs secreting the cognate CCR7 chemokine ligands CCL19 and CCL21. Of the CCR7 ligands, it has recently been shown that CCL21a is the major regulator of CCR7‐mediated SP thymocyte migration, and in its absence there is a failure in thymic tolerance that leads to autoimmunity.^43^ Importantly, the thymus medulla is also rich in a heterogeneous mixture of DCs, which play a key role in both negative selection and Foxp3^+^ T‐cell development. Interestingly, we recently showed that an explanation for the breakdown of thymic tolerance in *Ltbr^−/−^* mice is the reduction in the size of the intrathymic DC pool rather than loss of organized LTβR‐dependent mTEC, a finding that emphasizes the importance of DC for thymic tolerance.^32^ Whether this control of thymic DC maps to the ability of this receptor to regulate CCR7 ligand expression in thymic stroma^44^ is not clear, although the survival of at least some thymic DC is regulated via CCR7.^45^ In conclusion, the thymus medulla represents an important microenvironment for T‐cell development for several reasons. First, a period of medullary residency that follows positive selection in the cortex enables the thymus to impose central tolerance mechanisms on newly produced CD4^+^ and CD8^+^ thymocytes. Second, during their time within the medulla, mature thymocytes progressively acquire “egress competence” through a program of postselection maturation, which enables them to exit the thymus and enter the periphery.

## 3 REGULATORS OF THYMUS EMIGRATION

Although several studies have examined the time SP thymocytes spend within the thymus,^46^ most recent work indicates that a period of 4–5 days of residency follows progression to the SP thymocyte stage.^47^ In line with this period of medulla occupancy, SP thymocytes are developmentally heterogeneous. For example, early studies showed that HSA expression levels could be used to sequentially define different maturational stages within CD4^+^ SP thymocytes.^48^ HSA^hi^ cells were defined as “SM” cells still susceptible to tolerance induction, while HSA^lo^ cells were shown to be resistant to the induction of apoptosis following TCR stimulation. Thus, changes in the maturational status of the SP thymocytes can be revealed by their phenotypic properties. More recently, SP thymocyte heterogeneity has further been revealed using a variety of additional cell surface phenotypes, including the chemokine receptors CCR4, CCR7, and CCR9. Using this approach to analyze CD4^+^ SP thymocyte developmental heterogeneity, expression of CCR4 and CCR9 was shown to identify newly generated cells, with more mature cells having a CCR4^−^CCR7^+^CCR9^−^ phenotype.^49^, ^50^, ^51^ Additional phenotypic markers used to separate SP thymocytes on the basis of their developmental status include CD69, CD62L, and Qa2, although the relevance of expression levels of the latter in relation to maturational state has recently been questioned.^47^, ^52^, ^53^, ^54^ Most recently, Xing et al.^52^ redefined the progressive postselection maturation stages that occur in the medulla by analyzing the expression of CD69 and MHC Class I within SP4 and SP8 thymocytes. Importantly, this study was able to reveal 3 distinct populations within both SP4 and SP8 thymocytes that were distinct in terms of their responsiveness to TCR stimulation, as well as their thymus egress capabilities.^52^ Thus, comparative analysis showed that CD69^+^MHC Class I^−^ SP thymocytes were the least mature and hence these cells were termed SM. Next, CD69^+^MHC Class I^+^ SP thymocytes were termed mature 1 (M1) and shown to be proliferation‐competent following TCR stimulation, while CD69^−^MHC Class I^+^ cells were termed mature 2 (M2), which were shown to demonstrate both TCR‐induced proliferation and cytokine secretion competency.^52^ Importantly, M2 cells were also shown to express genes that control thymocyte egress, including the gene encoding the sphingosine‐1‐phosphate receptor 1 (S1PR1), which has been used as a marker of mature thymocytes in several studies.^52^, ^53^, ^55^ S1PR1 is a G protein‐coupled cell surface receptor which in the thymus mediates migration of S1PR1^+^ thymocytes toward a gradient of the lipid signaling molecule sphingosine‐1‐phosphate (S1P).^55^ The expression of several other important regulators including Forkhead box protein O1 (Foxo1) and Krüpple‐like factor 2 (Klf2) are also upregulated during SP thymocyte maturation.^56^, ^57^, ^58^ Significantly, CD62L and S1PR1 are both downstream targets of Klf2. Moreover, the expression of S1PR1/CD69 expression is linked, whereby CD69 possesses the capacity to bind and inhibit S1PR1 via internalization and degradation.^59^ Therefore, as SP thymocytes mature, they increase Foxo1 and Klf2 expression, which in turn up‐regulates CD62L as well as S1PR1 expression at the same time as CD69 is down‐regulated, such regulated patterns of expression likely act to limit the timing of thymic egress to mature thymocytes having undergone central tolerance events.^56^, ^57^, ^58^, ^60^

**Figure 2:**
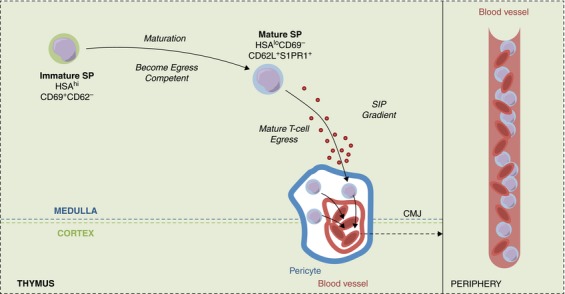
**Thymic T‐cell egress**. Following selection, SP thymocytes undergo maturation in the medulla where they develop from immature HSA^hi^CD69^+^CD62L^−^ to mature HSA^lo^CD69^−^CD62L^+^ SP thymocytes. This maturation enables SP thymocytes to express the sphingosine‐1‐phosphate (S1P) receptor 1 (S1PR1) and undergo thymus emigration. Mature thymocytes first migrate into the perivascular space (PVS) of blood vessels at the corticomedullary junction (CMJ) the space between pericytes and endothelial cells of the blood vessel, and then undergo reverse transendothelial migration into the blood

Through the careful examination of SP thymocyte heterogeneity described above, the process of thymocyte egress can be split into 3 separate stages (Fig. 2). The first of these steps involves the progressive maturation of medullary resident, postselection SP thymocytes that reach an egress competent stage as defined by their expression of S1PR1. This process enables S1PR1^+^ SP thymocytes to migrate toward an S1P gradient at least in part formed by the combined activity of pericytes and DCs surrounding blood vessels at the CMJ. This initial step is followed by a second phase in which SP thymocytes cross the basement membrane surrounding blood vessels to enter into the perivascular space (PVS), the region defined as the space between blood endothelial cells and surrounding pericytes. The final step comprises reverse transendothelial migration, in which mature SP thymocytes exit from the PVS and enter into the blood stream by migrating across thymic blood endothelium, enabling them to join the peripheral T‐cell pool as recent thymic emigrants (RTE). Currently, it is not fully understood how intrathymic microenvironments and particular thymic stromal cells influence each of these phases of the emigration process. Moreover, while the above findings suggest that emigration occurs via an ordered and linear “conveyor belt” mechanism in which only the most mature SP thymocytes are able to leave the thymus, earlier studies indicated that thymocytes may also leave the thymus as part of a “lucky dip” model.^47^, ^61^ While further work is required to examine stages in thymocyte egress and the factors that regulate this process, in the remainder of this review we summarize current knowledge on the known regulators of thymic exit.

## 4 THE S1P–S1PR1 AXIS

Perhaps the most well‐characterized mechanism of thymus emigration from the adult thymus involves expression of S1PR1 by mature thymocytes and the production of one of its ligands, S1P. Disruption of this axis via multiple means perturbs thymic output resulting in reduced T‐cells in the periphery and an intrathymic accumulation of mature thymocytes.^55^, ^62^, ^63^ Initial studies revealed the importance of this pathway for thymic egress through the use of the immunomodulator FTY720, a potent agonist of S1PR1, which prevents migration to S1P by inducing down‐regulation of the S1PR1. Thus, treatment of mice with FTY720 led to an intrathymic accumulation of mature thymocytes, and a reduction in peripheral T‐cell numbers in secondary lymphoid organs.^64^, ^65^, ^66^, ^67^, ^68^, ^69^

As S1PR1 ligation can cause receptor down‐regulation, intrathymic levels of S1P must be kept tightly restricted, such that free S1P is only available at functionally relevant levels in the close proximity of the blood vessels that represent the points of thymic exit into the S1P‐rich blood circulatory system. In this setting, it is critical for tight regulation of thymic emigration that S1P levels must remain sufficiently low within the rest of the thymic parenchyma to enable the formation of a suitable gradient for effective migration.^70^, ^71^ Several mouse models have revealed that within the thymus, multiple cell types regulate the S1P–S1PR1 axis by acting to establish and maintain the intrathymic S1P gradient. The mechanisms that regulate the S1P gradient within thymic tissues are therefore tightly regulated by a fine balance existing between the production and maintenance of high S1P levels at the site of exit, and regulation of low levels by degrading or dephosphorylating S1P within the surrounding thymic microenvironment.

### 4.1 Regulation of S1P levels

Maintaining high levels of S1P at the site of exit has primarily been attributed to the production of S1P by thymic pericytes. Two enzymes, sphingosine kinase 1 (Sphk1) and Sphk2, which catalyze the ATP‐dependent phosphorylation of sphingosine to generate S1P, are expressed by thymic pericytes that represent non‐epithelial cells ensheathing blood vessels at the sites of T‐cell exit.^71^ The essential role of thymic pericyte Sphk activity has been demonstrated by studies utilizing cell‐specific deletion of Sphk in thymic pericytes. Such conditional Sphk deletion resulted in an intrathymic accumulation of mature thymocytes and an accompanying peripheral T‐cell lymphopenia, both of which are indicative of disrupted thymocyte egress and thus highlights the crucial role of thymic mesenchyme in regulating T‐cell egress via the S1P pathway.^71^ In addition to thymic pericytes, additional nonepithelial stromal populations have been shown to regulate S1P‐dependent egress through the positive influence of S1P levels. Fukuhara et al.^72^ showed that thymic endothelium can regulate S1P‐dependent thymocyte egress through the expression of the S1P transporter spinster homolog 2 (Spns2). Thus, Spns2 is required for S1P release from endothelial cells, correspondingly deletion of Spns2 resulting in intrathymic accumulation of mature thymocytes consistent with an egress defect.^72^

Conversely, the maintenance of intrathymic S1P gradients can occur through the degradation or dephosphorylation of S1P to ensure low levels at the sites that the mature T‐cells migrate from. Such regulation can operate via the coordinated activity of both stromal and hematopoietic compartments. An example of stromal regulation is through the production of lipid phosphate phosphatase 3 (Lpp3), a dephosphorylating enzyme that inactivates S1P to maintain low intrathymic S1P levels promoting thymocyte egress.^73^ Deletion of Ppa2b, the gene encoding Lpp3, results in an intrathymic accumulation of mature thymocytes consistent with an egress defect. Interestingly deleting Ppa2b specifically on TECs or endothelial cells results in an equivalent intrathymic accumulation of mature thymocytes and therefore both TECs and endothelial cells regulate SIP‐dependent thymocyte egress through production of Lpp3.^73^ TECs and endothelial cells have also been shown to express S1P lyase (Sgpl), an enzyme that degrades S1P to maintain low levels of S1P to regulate T‐cell egress. However a recent study by Zamora‐Pineda et al.^74^ found that deletion of Sgpl in either TECs or thymic endothelial cells was not sufficient to cause a T‐cell egress defect. In fact it was only the deletion of Sgpl in bone marrow‐derived cells that resulted in reduced thymocyte egress and a concomitant intrathymic accumulation of mature SP thymocytes.^74^ These bone‐marrow derived cells included DCs as well as T‐cells themselves, suggesting additional cell types beyond the thymic microenvironment that are able to influence thymocyte egress via the S1P–S1PR1 pathway.^74^

S1P is generated through the phosphorylation of sphingosine which itself is synthesized from ceramide.^75^ Ceramide synthase 2 (Cers2) is a known regulator of sphingosine, acting to limit S1P levels via conversion of sphingosine into long‐chain ceramides.^76^ Recent studies have highlighted the essential role of Cers2 in the regulation of thymic egress. Rieck et al.^77^ revealed that Cers2‐deficient mice demonstrated an intrathymic accumulation of mature SP thymocytes as well as a reduction of SP4 and SP8 thymocytes within the blood and spleen. Further analysis revealed that the intrathymic and blood levels of S1P were increased and thus T‐cell egress was defective due to dysregulation of the S1P gradient, identifying Cers2 as an additional candidate to a growing list of regulators of S1P‐dependent T‐cell egress and thus highlighting the multifaceted aspect of the S1P pathway for thymic egress.^77^ Interestingly, the essential activity of Cers2 in the regulation of thymic egress was attributed to nonhematopoietic stromal cells, potentially including blood endothelium. Given the positioning of blood endothelial cells as the final cellular barrier between thymic microenvironments and the peripheral circulation, it raises the interesting proposition that blood endothelial cells act as a vital gatekeeper for thymic emigration.

## 5 CHEMOKINES

T‐cell development in the thymus involves the directed migration of cells through distinct thymic microenvironments. In relation to chemokine receptors and SP thymocyte migration, CCR7 plays a key role in entry of these cells to the medulla via the expression of its ligand CCL21 by mTEC.^23^, ^43^ For thymic egress, CCR7 has been shown to be dispensable for the exit of mature conventional and Foxp3^+^ regulatory αβT‐cells from the adult thymus.^23^, ^78^ In contrast, a role for CCR7 in egress from the neonatal thymus is supported by several observations. For example, Ccr7^−/−^ neonates show an increased frequency of thymocytes and decreased splenic T‐cell numbers.^79^ Moreover, injecting mice with reagents to selectively block either CCL19 or CCL21 function showed that, blocking CCL19 but not CCL21, resulted in increased thymocyte numbers and decreased splenic T‐cell numbers. While these observations are consistent with a role for CCR7–CCL19 in emigration in the neonatal period, it is important to note that a recent study analyzing Ccl19^−/−^ mice showed that CCL19 is required for normal splenic white pulp development, suggesting that the reduction in splenic T‐cell numbers is a direct consequence of defects in the spleen, and is not secondary to a thymus egress effect.^80^ Interestingly, and in support of this, no changes in the frequencies of SP thymocytes were noted in Ccl19^−/−^ neonates, and so the ligand requirements for CCR7‐mediated emigration from the neonatal thymus require further examination. However, it is also interesting to note that additional studies indicate the mechanisms involving CCR7 that control egress from the neonatal and adult thymus may be different. For example, neonatal Aire^−/−^ mice, which were reported to have reduced intrathymic CCR7‐ligand expression, also show evidence of impaired thymocyte egress.^81^ This study also indicated that while the thymic S1P–S1PR1 pathway is functional at the neonatal stage, it is not sufficient to correct for the defect in CCR7‐dependent egress.^81^ However, beyond 3 weeks of age in Aire^−/−^ mice, S1PR1 underwent significant compensatory upregulation on mature thymocytes, which alleviated the T‐cell egress defect seen in neonatal mice.^81^ Thus, accumulating evidence indicates that CCR7 may act in concert with other regulators of thymic egress in a manner that is influenced by neonatal/adult time periods.

The chemokine receptor CXCR4 has also been suggested to play a role in thymus emigration. However, until recently, this has been difficult to directly examine in vivo due to the embryonic lethality of mice lacking CXCR4, and its ligand CXCL12.^82^, ^83^, ^84^, ^85^ Consequently, many experimental approaches that have been used to address the role of CXCR4 in mature SP thymocyte migration involve in vitro thymus organ cultures and/or the pharmacologic inhibition of CXCR4–CXCL12 function.^83^, ^84^ However, using a Cre‐mediated stage‐specific approach to delete CXCR4 expression from the DP thymocyte stage, the role of CXCR4 in thymic emigration was recently analyzed in the steady state thymus in vivo. Interestingly, analysis of CD4^cre^CXCR4^flox^ mice found no abnormalities in SP thymocyte development or egress, suggesting that CXCR4 is dispensable for these processes.^86^ Importantly, this lack of requirement for CXCR4 in SP thymocyte migration also correlated with the rapid termination of CXCR4 following the initiation of positive selection, and the predominant expression of CXCL12 in the thymic cortex and not the medulla.

While the above studies indicate the differential requirement for certain chemokines in thymic emigration, the cell types that express these molecules, and the mechanisms that control their production in thymic stroma, are not fully understood. However, pioneering studies by Boehm et al.^30^ showed that LTβR, a TNFRSF member that regulates chemokine expression in lymphoid tissues plays an essential role in controlling thymic egress.^87^, ^88^ Thus, adult Ltbr^−/−^ mice were shown to have an intrathymic accumulation of mature SP4 and SP8 thymocytes, as well as altered medullary organization and mTEC numbers.^30^ Whether the requirement for LTβR in thymic egress maps to its ability to control intrathymic expression of chemokines remains unclear. Interestingly however, TECs express the CCR7‐ligands CCL21 and CCL19, which are known targets of LTβR signaling, and LTβR‐deficient mice have been showed to have a reduction in CCL21^+^ mTEC.^44^, ^87^ Relevant to this, as CCR7 is not required for thymus emigration in the adult, it is perhaps likely that LTβR regulates T‐cell egress via mechanisms additional to its control of CCR7 ligand availability.

## 6 THE TYPE 2 IL‐4R AND THYMIC EMIGRATION

In an attempt to identify novel regulators of thymus emigration, we examined the thymic architecture of mice carrying deletions in genes identified as being expressed by TEC via microarray analysis. Specifically, we investigated the intrathymic positioning of mature SP4 and SP8 thymocytes, and concentrated on mouse strains where the typical random distribution of these cells within thymic medullary areas was altered. We saw that in Il4ra^−/−^ mice, the thymus medulla contained large mTEC‐free areas filled with SP thymocytes,^89^ and the thymus was enriched in the most mature CD69^−^CD62L^+^ SP4 thymocyte subset. Further examination showed these structures to be enlarged PVS that were surrounding thymic blood vessels. Interestingly, IL‐4Rα is a component of 2 cytokine receptors. Paired with the common gamma chain, it forms the Type 1 IL‐4R complex on lymphocytes with binds IL‐4. In contrast, when IL‐4Rα is complexed with IL‐13Rα1 on stromal cells, it forms the Type 2 IL‐4R complex that binds both IL‐4 and IL‐13. mTECs were found to express all Type 2 IL‐4R components, and the intrathymic accumulation of SP thymocytes was found to map to IL‐4Rα expression by thymic stroma. Thus, Type 2 IL‐4R expression by the thymic microenvironment represents an important regulator of SP thymocytes where it acts as a regulator of thymic egress. When examining whether the role of IL‐4Rα in this process was connected to the known role for the S1P–S1PR1 axis, we found that cell surface levels of S1PR1 and CD69 on mature thymocytes in WT and Il4ra^−/−^ mice were comparable, suggesting that intrathymic S1P levels were not substantially altered. Moreover, treatment of both WT and Il4ra^−/−^ mice with FTY720 resulted in an intrathymic retention of SP thymocytes, indicating that S1PR1‐mediated migration remained active in the thymus of Il4ra^−/−^ mice. While these findings suggest that the requirement for the Type 2 IL‐4R in thymus emigration is distinct to the involvement of S1P–S1PR1, its mechanism of action is unclear. Relevant to this is that the thymus accumulation seen in Ltbr^−/−^ mice does not appear to involve accumulation within thymic PVS, making it perhaps unlikely that the IL‐4Rα axis is directly regulated by LTβR. Interestingly, triggering Type 2 IL‐4R signaling in thymic stroma with either IL‐4 or IL‐13 induced the expression of a broad array of chemokines including CCL21, one of the ligands for CCR7 that has been implicated in thymus emigration in the neonate.^79^ While further work is required to examine the role of IL‐4Rα in thymic egress, its role in this process was shown to map to the provision of the type 2 cytokines IL‐4 and IL‐13 by a thymic‐resident subset of CD1d‐resctricted iNKT‐cells, providing a cellular mechanism for its action. Finally, that innate‐like iNKT‐cells play a role in controlling the emigration of conventional αβT‐cells from the thymus provides a further example of how innate‐like cells influence thymus function via cellular crosstalk in the medulla.^90^

### 6.1 T‐cell intrinsic regulators of T‐cell egress

As well as regulation of T‐cell egress occurring via T‐cell extrinsic regulation, T‐cell intrinsic pathways have been identified that are essential for T‐cell egress. The protein kinase Mst1 forms a complex with RAPL to activate Mst1 kinase, which regulates lymphocyte polarization and adhesion stimulated by chemokines and TCR signaling. In the context of T‐cell egress Dong et al.^91^ showed that MST1 plays an essential role in regulating T‐cell egress. Mst1^−/−^ mice exhibit an intrathymic accumulation of mature SP thymocytes as well as reduced lymphocytes both in the blood and peripheral tissues.^91^ Interestingly through the use of p56Lck^cre^Mst1^fl^ mice, the role of Mst‐1 was shown to be T‐cell intrinsic as these mice also had an intrathymic accumulation of mature SP4 and SP8 thymocytes.^91^ Mst1^−/−^ thymocytes have impaired chemotactic response to chemokines but not S1P suggesting the regulation of T‐cell egress by Mst1 is S1P‐independent, again suggesting that active thymic emigration relies upon a fine‐tuned interplay between multiple regulators of T cell migration.^91^ The role of Mst2 was also identified through the use of Mst1^−/−^Mst2^−/−^ double knockout mice, which exhibited an exacerbated intrathymic accumulation that was of greater magnitude than the Mst1^−/−^ thymus implicating Mst2, as well as Mst1, in regulating T‐cell egress.^92^

The actin regulator Coronin‐1A (Coro1a) has additionally been shown to be essential for normal thymic egress. The mouse strain Cataract Shionogi (CTS) was initially reported to have a T‐cell egress defect, exhibiting an intrathymic accumulation of SP thymocytes within the perivascular space and reduced RTE in the periphery.^93^ It was later found that the CTS strain phenotype was caused by a point mutation in the gene encoding Coro1a.^94^ Interestingly mice deficient for Coro1a have a reduction in peripheral T‐cells however this phenotype is also accompanied by an intrathymic loss of mature SP thymocytes due to impaired survival and thus investigation of the importance of Coro1a is complicated by this phenotype. However the CTS strain which carries the peripheral T‐cell deficiency (Ptcd) locus and thus have the point mutation in the *Coro1a* gene displayed normal cell survival of mature thymocytes and subsequently this revealed a block in T‐cell egress as measured by an intrathymic accumulation of mature SP thymocytes as well as the reduction of SP thymocytes within the periphery.^93^, ^94^ Surprisingly, despite these SP thymocytes expressing normal S1PR1, their ability to migrate toward S1P, as well as other chemokines, was significantly impaired due to defective actin remodelling.^94^ This intrathymic accumulation in the absence of impaired cell survival as well as the inability to migrate toward S1P and chemokines such as CCL21 highlights a key role of Coro1a in regulating T‐cell thymic egress.

## 7 CONCLUDING REMARKS

The regulation of thymus‐dependent αβT‐cell maturation concludes with the release of those mature, functionally self‐tolerant T‐cells that have survived the rigors of intrathymic selection events into the systemic circulatory system. Notably the development of thymocytes follows a strictly controlled pathway of maturational hurdles sequentially characterised by thymus entry and T‐cell specification, positive selection, central tolerance enforcement and finally acquisition of egress competency. The developmental transition of T‐cells along this course is dictated via the coordinated migration of thymocytes through highly specialized subcompartments of the thymus defined by a diverse mixture of both hematopoietic and stromal cell types. Despite the importance of this process to the generation of a sufficiently diverse repertoire of αβT‐cells capable of providing protection against pathogenic challenge and tumor formation, the precise cellular and molecular pathways that dictate this process remain incompletely understood. For instance, although several G‐protein coupled receptor‐associated pathways, including both chemokine and S1P interactions have been implicated in the regulation of thymic emigration, the combinatorial effect of such pathways remains unclear. In particular determining the precise balance of those signals that act to retain thymocytes within the thymic medulla to ensure sufficient screening of T‐cells for self‐reactivity versus those that positively drive thymic egress will be critical to advance our understanding of how the pressures of constantly replenishing the peripheral T‐cell repertoire are balanced against the need to ensure self‐tolerance via medullary dwell‐time. Moreover, whilst it is important to understand how these processes operate in the steady state, how the balance of intrathymic T‐cell retention and egress may be altered following acquired peripheral T cell lymphopenia and the impact that this may have on central tolerance raises important questions. In summary, whilst the mechanisms regulating thymic αβT‐cell maturation and emigration have begun to be unraveled, further work defining these processes will have important implications for the future development of routes to manipulate T‐cell tolerance and seeding of the peripheral T‐cell pool.

## DISCLOSURES

The authors declare no conflicts of interest.
